# Improving Potato Stress Tolerance and Tuber Yield Under a Climate Change Scenario – A Current Overview

**DOI:** 10.3389/fpls.2019.00563

**Published:** 2019-05-14

**Authors:** Keshav Dahal, Xiu-Qing Li, Helen Tai, Alexa Creelman, Benoit Bizimungu

**Affiliations:** Fredericton Research and Development Centre, Agriculture and Agri-Food Canada, Fredericton, NB, Canada

**Keywords:** yield, stress tolerance, potato, high CO_2_, high temperature, drought, salinity, photosynthetic yield potential

## Abstract

Global climate change in the form of extreme heat and drought poses a major challenge to sustainable crop production by negatively affecting plant performance and crop yield. Such negative impact on crop yield is likely to be aggravated in future because continued greenhouse gas emissions will cause further rise in temperature leading to increased evapo-transpiration and drought severity, soil salinity as well as insect and disease threats. This has raised a major challenge for plant scientists on securing global food demand, which urges an immediate need to enhance the current yield of major food crops by two-fold to feed the increasing population. As a fourth major food crop, enhancing potato productivity is important for food security of an increasing population. However, potato plant is highly prone to high temperature, drought, soil salinity, as well as insect and diseases. In order to maintain a sustainable potato production, we must adapt our cultivation practices and develop stress tolerant potato cultivars that are appropriately engineered for changing environment. Yet the lack of data on the underlying mechanisms of potato plant resistance to abiotic and biotic stress and the ability to predict future outcomes constitutes a major knowledge gap. It is a challenge for plant scientists to pinpoint means of improving tuber yield under increasing CO_2_, high temperature and drought stress including the changing patterns of pest and pathogen infestations. Understanding stress-related physiological, biochemical and molecular processes is crucial to develop screening procedures for selecting crop cultivars that can better adapt to changing growth conditions. Elucidation of such mechanism may offer new insights into the identification of specific characteristics that may be useful in breeding new cultivars aimed at maintaining or even enhancing potato yield under changing climate. This paper discusses the recent progress on the mechanism by which potato plants initially sense the changes in their surrounding CO_2_, temperature, water status, soil salinity and consequently respond to these changes at the molecular, biochemical and physiological levels. We suggest that future research needs to be concentrated on the identification and characterization of signaling molecules and target genes regulating stress tolerance and crop yield potential.

## Introduction

Global climate change poses a major challenge to sustainable crop production. Global climate change has affected weather patterns resulting in extremes of heat, drought, frequent frost and snow fall in high altitudes (IPCC, 2014). The sub-optimal growth conditions associated with global warming and climate change negatively impact plant growth, survival and crop yield (Lesk et al., 2016). Such negative impact on plant performance and crop yield is likely to be aggravated in future because continued greenhouse gas emissions will intensify crop plant’s exposure to abiotic and biotic stresses (DeLucia et al., 2012; IPCC, 2014). Climate scientists have projected that the current ambient CO_2_ concentration of 380 ppm will double to ca. 700 ppm by the end of the 21st century (IPCC, 2007), which is likely to be coupled with a rise in the global air temperature by 0.3 to 4.8°C (IPCC, 2014). The predicted increase in the atmospheric temperature may increase the evapo-transpiration water loss causing soil water limitation and agricultural drought (Hatfield et al., 2011; Vandegeer et al., 2012). Climate change is also predicted to increase soil salinity particularly in the coastal regions through sea level rise and salt water intrusion (Rahmstorf, 2007; Dasgupta et al., 2009). Recent study suggests that the geographical distributions of pest and pathogens, and their interactions with plant hosts, including changes in host susceptibility, will be affected by changing climate (Elad and Pertot, 2014). Thus, while the anticipated increase in the atmospheric CO_2_ level may enhance yield potential in certain crop species (Deryng et al., 2016) the yield losses due to high temperature and water deficit may surpass the benefit achieved by any increase in CO_2_ (Lobell and Gourdji, 2012). Moreover, the sub-optimal growth conditions are occurring at a time of predicted 30% increase in the world population by 2050 (United Nations Department of Economic and Social Affairs, 2011). This has created a global challenge concerning food security, which urges that the yield of major food crops needs to be increased two-fold over the next 50 years to fulfill the nutritional requirements of the increasing population (Murchie et al., 2009). The projected increase in food demand is further complicated by decrease in the total area of agricultural land due to desertification and urbanization, and increase in the food grain demand for animal nutrition and biofuel generation (Murchie et al., 2009; Zhu et al., 2010).

**FIGURE 1:**
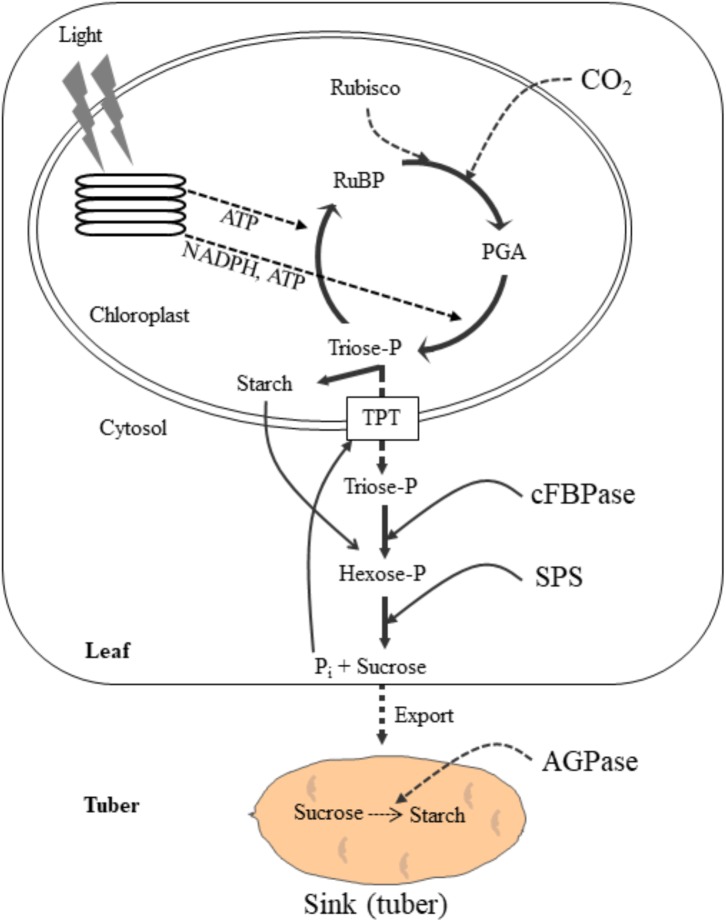
A schematic diagram illustrating the photosynthetic carbon assimilation and its export to the potato tuber sink. In the chloroplast, light harvesting complexes absorb sunlight and generate ATP and NADPH through photosynthetic electron transport chain. The ATP and NADPH are then consumed by Calvin cycle to assimilate carbon to simple carbohydrate, triose phosphates (triose-P). Triose-P is either used to synthesize starch in the chloroplast or exported to cytosol in exchange for Pi through triose-P/P_i_ translocator. In the cytosol, the triose-P is then converted to sucrose via hexose phosphates using key sucrose biosynthetic enzymes, cFBpase and SPS. Sucrose is then transported to the sink (tuber), where it is converted to starch by AGPase. The synthesis of sucrose in the cytosol generates P_i_ which is exported to the chloroplast in the exchange for triose-P. Any stress that inhibit sucrose synthesis and its transport to sink (tuber) results in the feedback inhibition of photosynthesis due to P_i_ regeneration limitation. The broken arrows indicate the stress sensitive processes. RuBisCO, ribulose-1,5-bisphosphate carboxylase/oxygenase; RuBP, ribulose-1,5-bisphosphate; TPT, triose phosphate translocator; cFBPase, cytosolic fructose bisphosphatase; SPS, sucrose phosphate synthase, AGPase: ADP glucose pyrophosphorylase. Modified from Hüner et al. (2016).

The global potato production is estimated to be 382 million tons in 2014 ranking first highest produced non-cereal food crop and the fourth highest produced crop worldwide after wheat, corn and rice (FAOSTAT, 2017). Potato is cultivated in over 100 countries feeding over a billion people worldwide. It is a rich source of carbohydrates and provides other essential nutrients, such as dietary fiber, vitamins, minerals, protein and antioxidants (Bach et al., 2012). Hence, enhancing potato crop productivity can contribute to fulfill the nutritional requirements of the rising population (Birch et al., 2012). Potato is mainly grown for its tubers. Synthesis of carbohydrates through photosynthesis in the source leaves, translocation of photosynthetic end product, sucrose, to the stolon and conversion of sucrose to starch in the stolon are key physiological process for potato tuber initiation and growth (Figure 1). The effective coordination among these processes determines tuber productivity and quality. During photosynthesis, photosynthetic electron transport chain generates ATP and NADPH, which are then consumed by Calvin cycle to assimilate CO_2_ to carbohydrates (Stitt et al., 2010; Rochaix, 2011; Foyer et al., 2012). The photosynthetic end product, sucrose, is then translocated into the underground stem via phloem loading and converted into starch (Figure 1). Any stresses that have negative effects on these processes may substantially inhibit tuberization and tuber growth resulting in lower tuber yield and quality. Since the potato tuber is chiefly composed of photoassimilates, mainly starch, an increase in tuber yield can be expected through stimulation of photosynthetic CO_2_ assimilation and translocation of the photosynthetic end product to the underground stem. Major abiotic stresses namely, high temperature, drought, soil salinity and nutrient stresses adversely affect these processes and substantially curtail plant growth, tuberization, tuber bulking, and hence tuber yield and quality (Minhas, 2012; Wang-Pruski and Schofield, 2012). The magnitude of yield loss due to these stresses, however, depends on the duration, severity and plant growth stage (Evers et al., 2010). Early stress is most detrimental to tuberization, bulking and tuber yield as a result of reduced rates of carbon assimilation and decreased partitioning of assimilates to tubers (Obidiegwu et al., 2015). It has been predicted that potato yield will decline substantially by 2055 due to global warming and drought (Holden et al., 2003). In another study Hijmans (2003) anticipates that world potato production will decline by 18–32% in the projected period of 2040–2069 as a consequence of biotic and abiotic stresses associated with climate change. Thus, in order to improve potato yield, we need to identify best production practices and develop new potato cultivars that best fit in the predicted climate change. Yet the lack of data on the precise mechanisms of plant resistance to abiotic stress and the subsequent ability to predict future outcomes constitute a major knowledge gap. This review paper first describes processes of enhancing crop yield through improved energy conversion efficiency into biomass and crop yield followed by some important abiotic stresses impacting this efficiency. The main focus will be on how these abiotic factors impact potato growth, development and yield, and possible adaptation strategies to combat these stresses.

## Enhancing Potato Productivity Through Improved Photosynthetic Yield Potential

Since the mid-1950s the increased application of pesticides, fertilizers and irrigation water as well as genetic improvement have mainly contributed to the enhanced yield of major food crops (Murchie et al., 2009). The slower yield increase of principal food crops since the last decade suggests that the yield enhancement due to increased use of agricultural inputs and improved cultivation practices has reached a maximum theoretical limit (Zhu et al., 2008, 2010). Consequently, the additional increase in the yield of major crops can only be expected through improving genetic yield potential that is, the crop yield that a plant can achieve per unit ground area under optimum growth conditions in the absence of biotic and abiotic stresses (Zhu et al., 2010; Ort et al., 2014). The genetic gain in potato, particularly yield, has been low and is in need for improvement (Jansky, 2009; Hirsch et al., 2013). The maximum potential yield is determined by a number of yield components associated with photosynthesis. They are (i) amount of incident solar radiation (ii) light interception efficiency, by which photosynthetic pigments intercept photosynthetically active radiation (PAR, 400–700 nm) (iii) photosynthetic efficiency, through which the intercepted light energy is converted to biomass (iv) partitioning efficiency, by which the biomass energy is partitioned into seeds/tubers also known as harvest index (Long et al., 2006; Amthor, 2007; Zhu et al., 2008, 2010). After 1960’s green revolution, the light interception efficiency and the energy partitioning efficiency have approached the plateau due to release of new cultivars and intensive use of agricultural inputs. Thus, further enhancement in yield potential can only be obtained by improving photosynthetic light conversion efficiency (Ort et al., 2014). This notion has been supported by recent studies, which revealed that improving photosynthetic efficiency significantly increases wheat and rye grain yield (Dahal et al., 2014b), tobacco biomass (Kromdijk et al., 2016) and tobacco seed yield (Dahal and Vanlerberghe, 2018b). Although photosynthesis is central to convert solar incident energy into biomass and crop yield, improving photosynthetic efficiency has received little research priority in enhancing crop yield (Long et al., 2006; Zhu et al., 2010).

**FIGURE 2:**
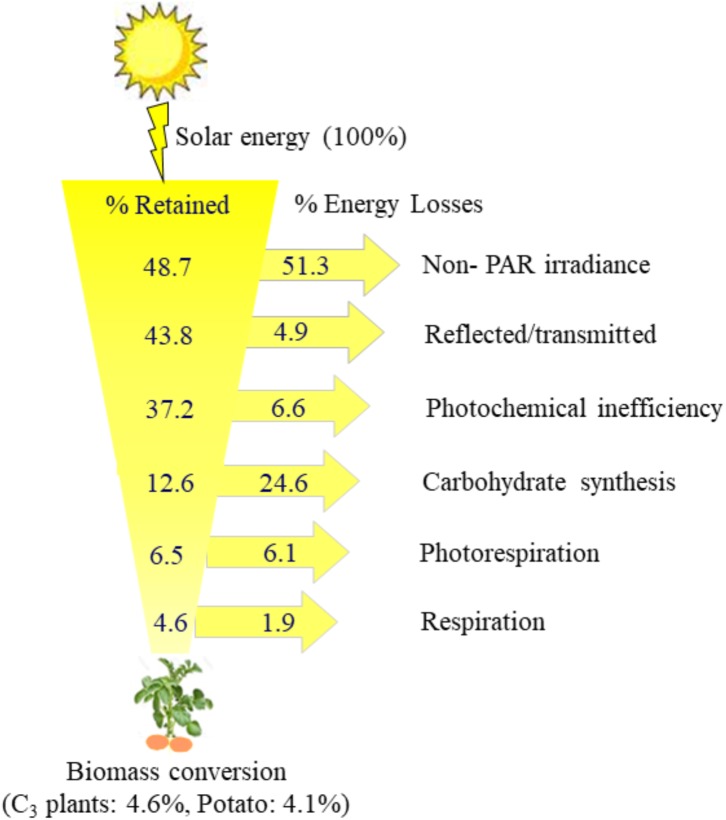
A simplified model for the processes involved in the conversion of solar incident energy to final plant biomass and associated energy losses. Out of 100% solar incident energy the C_3_ photosynthetic organisms are capable of converting only 4.6% to final plant biomass, suggesting a maximum theoretical energy conversion efficiency of 0.046. Most portion of solar energy is lost resulting in considerable decrease in energy conversion efficiency between the solar energy and final plant biomass. Redrawn from Zhu et al. (2008, 2010); and modified based on Dahal et al. (2014a,b).

The processes of solar incident energy conversion to plant biomass through C_3_ photosynthesis and associated energy losses are illustrated in Figure 2. Since about 51.3% of the total solar energy striking leaf surface is outside of the 400–700 nm range of PAR, photosynthetic pigments intercept only remaining 48.7% of the total solar incident energy. However, photosynthetic organisms reflect or transmit about 4.9% of total solar incident energy as they weakly absorb in the green region of visible spectrum. This leaves only 43.8% of the total solar energy available for absorption by chlorophylls in the leaf. Leaf chlorophylls absorb maximally in the blue and red regions of PAR with 400 and 700 nm wavelengths, respectively. The reaction centers in the Photosystem I (PSI) and PSII drive photochemistry with the energy level of red photons only. Consequently, the energy absorbed at blue photon needs to be converted to the lower energy level of red photons resulting in heat dissipation of 6.6% of the intercepted solar energy, leaving only about 37.2% of the initial solar energy (Figure 2). The assimilation of one CO_2_ molecule into carbohydrate by Calvin cycle requires 2NADPH and 3ATP molecules (Zhu et al., 2008). The absorption of four moles of photons by chlorophyll molecules will generate one molecule of NADPH through linear electron transport chain, which is coupled to translocation of six protons into the thylakoid lumen. The synthesis of 1ATP molecule require 4 protons. Thus, absorption of 8 moles of photons are required to generate 2NADPH and 3ATP molecules to assimilate one CO_2_ molecule into carbohydrate. The eight moles of red photons contain 1388 kJ energy whereas one-sixth of a glucose molecule (1C carbohydrate unit), contains only 477 kJ energy (Zhu et al., 2008). Thus, the minimum energy loss between photons absorbed by reaction centers and photosynthetic linear electron transport chain to carbohydrate assimilation is, 1 – (477/1388), which represents a loss of 24.6% of the total incident solar energy, leaving about 12.6% of the total energy (Zhu et al., 2008). In C_3_ plants, photorespiration and respiration result in the loss of fixed carbon causing 6.1 and 1.9% expense of total energy. Thus, it is estimated that out of the total incident solar energy striking the leaf surface only about 4.6% is conserved to plant biomass, suggesting a maximum theoretical energy conversion efficiency of 0.046 in C_3_ plants (Figure 2). This estimate, however, does not include energy consumed in N and S reduction which needs to be taken in to account as they also consume photosynthetically generated electrons (Foyer and Noctor, 2002).

Potato tuber is chiefly composed of photoassimilates mainly starch. Hence, an enhancement in tuber yield can be obtained through stimulation of photosynthetic carbon fixation and its translocation to underground stem. Slattery and Ort (2015) have suggested an energy conversion efficiency between 0.028 and 0.041 for eight different C3 plants, with potato as the best with an energy conversion efficiency of 0.041. Since current energy conversion efficiency of potato is less than the maximal theoretical limit expected for C_3_ plants (Figure 2), there is a great potential to enhance potato tuber yield through improved photosynthetic efficiency. Potato cultivars exhibit a considerable difference in rates of photosynthesis, which can be used effectively in breeding new cultivars with a higher photosynthetic yield potential (Cieply, 1976). These differences in rates of photosynthesis can be used as a physiological marker for screening potato germplasms for higher tuber yield potential. It should be, however, considered that the canopy level net CO_2_ assimilation, duration of photosynthetic activity and transport of photoassimilates determines tuber size (Frier, 1975). Several studies suggest that increase in atmospheric CO_2_ will stimulate photosynthetic yield potential. However, one should also note that the CO_2_-stimulation of rate of photosynthesis is substantially offset by higher than optimal temperature, drought and soil salinity. Thus, future breeding programs should focus on screening potato cultivars that have potential to maintain higher CO_2_ assimilation rates under abiotic stress associated with global warming and climate change. Following section will discuss how elevated CO_2_, high temperature, drought and soil salinity impact on maximum potential yield with particular focus on potato and photosynthetic efficiency.

## Rising Atmospheric Co_2_

In a comprehensive review of several crop species, Ainsworth and Long (2005) reported that the predicted rise in atmospheric CO_2_ concentrations will benefit most C_3_ plants. Elevated CO_2_ is expected to stimulate photosynthesis and enhance light use efficiency into biomass and crop yield as well as improve water use efficiency (WUE) through closing stomata and preventing transpirational water loss (Ainsworth and Rogers, 2007; Dahal et al., 2014b; Dahal and Vanlerberghe, 2018a). Short-term exposure of C_3_ plants from ambient to elevated CO_2_ immediately stimulate the rates of photosynthesis (Cheng et al., 1998; Long et al., 2004; Ainsworth and Rogers, 2007; Dahal et al., 2012). This CO_2_ stimulation of rates of carbon assimilation in C_3_ plants is accounted for by two reasons. First, Ribulose-1,5-bisphosphate carboxylase/oxygenase (RuBisCO) is limited by CO_2_ substrate at current ambient CO_2_ concentrations of 380 ppm, the value close to the K_m_ (CO_2_) for RuBisCO (Long et al., 2004; Tcherkez et al., 2006). Therefore, an immediate increase in RuBisCO carboxylation velocity can be achieved through increased availability of CO_2_ substrate for RuBisCO following exposure to elevated CO_2_. Second, high CO_2_ competitively suppresses rates of photorespiratory carbon loss since CO_2_ is a competitive inhibitor of the RuBisCO oxygenation reaction (Long et al., 2004). However, during long-term growth at elevated CO_2,_ C_3_ plants may exhibit feedback inhibition of initial photosynthetic stimulation achieved following short-term exposure. The feedback inhibition of photosynthesis is accounted for by accumulation of non-structural carbohydrates in the source leaves (Stitt and Quick, 1989; Foyer, 1990). For details, the readers are suggested to read the theoretical model of the photosynthetic response of C_3_ plants to CO_2_ proposed by Farquhar et al. (1980). Up on long-term growth at elevated CO_2_, and when measured at higher C_i_, photosynthetic rate is usually limited either by the capacity of photosynthetic electron transport chain to supply ATP and NADPH to regenerate Ribulose-1,5-bisphosphate (RuBP) or by the capacity of starch and sucrose synthesis to utilize triose phosphates and regenerate P_i_ (Yang et al., 2016). The P_i_ regeneration-limited photosynthesis is governed by the balance between the source leaves and sink strength to supply and consume the carbon (Arp, 1991; Drake et al., 1997). The initial stimulation of carbon assimilation by elevated CO_2_ results in the accumulation of carbohydrates due to limited sink capacity to utilize carbohydrates and concomitant decreased carbon export to the sinks (Kramer, 1981; Arp, 1991; Drake et al., 1997). This results in a short-term depletion in stromal P_i_ due to decreased utilization of phosphorylated intermediates. Consequently, ATP synthesis in the chloroplast is inhibited leading to a decrease in the rate of phosphoglyceric acid (PGA) conversion to triose phosphate and eventually feedback limitation of photosynthesis (Sharkey and Vanderveer, 1989; Stitt and Quick, 1989). The feedback limitation of photosynthesis is also suggested to be associated with the downregulation of the key regulatory photosynthetic gene expression and corresponding enzyme activities (Harley and Sharkey, 1991; Drake et al., 1997; Moore et al., 1999).

The effects of rising CO_2_ concentrations on the growth and development of potato plants have been studied under growth chambers (Cao et al., 1994; Mackowiak and Wheeler, 1996; Wheeler and Tibbitts, 1997; Fleisher et al., 2008; Kaminski et al., 2014), in the greenhouse (Goudriaan and de Ruiter, 1983), open top chambers (Sicher and Bunce, 1999, 2001; Donnelly et al., 2001; Lawson et al., 2001; Finnan et al., 2002; Katny et al., 2005) and under free air carbon dioxide enrichment (FACE) systems (Miglietta et al., 1997, 1998). Most studies revealed that the growth and development of potato plants under high CO_2_ enhanced tuber yield by stimulating photosynthetic light use efficiency, and improved WUE through closing stomata and preventing transpirational water loss (Table 1). In a FACE experiment elevated CO_2_ stimulated potato tuber yield by 34 and 53%, and WUE by 70 and 67% in 1998 and 1999, respectively (Magliulo et al., 2003). The open top chamber experiment revealed about 40% increase in “Bintje” tuber yield at elevated CO_2_ of either 550 or 680 μmol CO_2_ mol^-1^ compared to at ambient CO_2_ (Donnelly et al., 2001). This stimulation was mainly associated with increased average tuber weight with tuber number having minimal effect. Similarly, in both controlled environment and field experiment, potato plants exhibited 30% yield stimulation when grown and developed at elevated CO_2_ than at ambient CO_2_ (Mackowiak and Wheeler, 1996). The increase in tuber yield was associated with either enhanced leaf area index (Donnelly et al., 2001) or rates of net carbon fixation per unit leaf area following growth at elevated CO_2_ (Schapendonk et al., 2000). A recent growth chamber study revealed an enhancement of WUE by 89 and 147% when grown at 700 and 1000 μmol CO_2_ mol^-1^, respectively, in comparison to growth at ambient CO_2_ (Kaminski et al., 2014). This enhancement was associated with stimulation of CO_2_ assimilation by 62 and 43%, with concomitant inhibition of stomatal conductance by 21 and 43% and leaf transpiration rates by 19 and 40% at 700 and 1000 μmol CO_2_ mol^-1^, respectively, compared to at ambient CO_2_ (Kaminski et al., 2014).

**Table 1:** Summary of the effects of elevated CO_2_, drought, high temperature and salinity on physiological, morphological and molecular characteristics of potato and acclimation/adaptation strategies.

	Responses to change in the growth environment			Acclimation/ Adaptation strategies
	Morphological	Physiological	Molecular	
High CO_2_	• Increased plant biomass	• Increased photosynthesis	• Down-regulation of key	• Stomatal closure
				• Resource remobilization particularly nitrogen in plant
	• Increased tuber yield	• Changed respiration rates	• photosynthetic enzyme activities particularly RuBisCO.	
		• Changed photorespiration rates		
				• Decreased RuBisCO activity
	• Increased LAI			
		• Reduced stomatal conductance		
	• Increased leaf DM content			
		• Reduced transpiration rates		
		• Increased water use efficiency		
		• Accumulation of non-soluble carbohydrates.		
		• Increased leaf nitrogen content		
Drought	• Reduced plant growth	• Declined photosynthesis	• Up-regulation of drought-responsive gene expression	• Stomatal closure
		• Changed respiration rates		• Increased root to shoot ratio.
	• Reduced plant biomass			
				• Increased ABA synthesis
			• Down-regulation of key photosynthetic gene expression	
		• Reduced chlorophyll content		• Increased osmolyte content.
	• Reduced tuber yield	• Reduced internal CO_2_ concentrations	• Reduced activities of key photosynthetic enzymes.	• Increased synthesis of drought-related proteins,
	• Reduced stolon and tuber number	• Reduced transpiration rates		
			• Increased activities of starch degrading enzymes	• Narrower leaf with hair.
		• Starch mobilization to reducing sugars		
				• Increased anti-oxidant
	• Reduced LAI			
		• Tuber develops sugar ends		
				• Increased glycoalkaloids
	• Increased leaf DM content			
		• Tuber develops malformations (hollow heart, cracking and secondary growth)		
	• Shorter plant height			
	• Increased root to shoot ratio			
		• Tuber develops internal brown spot		
	• Delayed tuberization			
	• Early senescence			
High temperature	• Reduced plant growth	• Declined photosynthesis	• Increased activities of starch degrading enzymes	Increased synthesis of heat-shock proteins
	• Reduced tuber yield	• Reduced photosystem II activity		
				• Increased transpiration
	• Increased leaf DM content	• Reduced sucrose translocation to tubers.		
	• Decreased tuber DM content	• Starch mobilization to reducing sugars		
		• Tuber develops sugar ends		
	• Delayed tuberization			
		• Tuber develops malformations (hollow heart, cracking and secondary growth)		
		• Tuber develops necrosis		
		• Tuber develops field sprouting		
Salinity	• Reduced plant emergence	• Declined photosynthesis	• Increased activity of transmembrane transport proteins involved in Na^+^ transport to vacuole	• Stomatal closure
		• Reduced transpiration rates		• Increased ABA
	• Reduced root length and volume			• Increased proline
				• Increased Na^+^ transport across the tonoplast in exchange for H^+^
		• Reduced leaf water content		
	Early haulm senescence			
			• Reduced activities of nitrate reductase	
				• Increased activity of antioxidant enzymes; ascorbate peroxidise, catalase, glutathione reductase and hydrogen peroxide
		• Reduced leaf osmotic potential		
			• Down-regulation of genes coding for Photosystem I, Photosystem II and chlorophyll synthesis proteins	
	• Reduced shoot biomass	• Increased total soluble solids		
		Increased lipid peroxidation		
	• Reduced tuber growth			
				• Change in chloroplast ultra-structure
		• Increased leaf carbohydrate content		
	• Decreased tuber DM content			
		• Reduced tuber nitrogen content	• Change in gene expression related to carbohydrate and amino acid metabolism	
	• Reduced tuber number			
	• Reduced tuber yield			


*LAI, Leaf area index; DM, dry matter. Data were obtained from: High CO_*2*_ (Sage et al., 1989; Mackowiak and Wheeler, 1996; Sicher and Bunce, 2001; Donnelly et al., 2001; Vandermeiren et al., 2002; Magliulo et al., 2003; Katny et al., 2005; Kaminski et al., 2014); Drought (Dalla Costa et al., 1997; Chen and Murata, 2008; Evers et al., 2010; Watanabe et al., 2011; Albiski et al., 2012; Wang-Pruski and Schofield, 2012; Obidiegwu et al., 2015; Zarzynska et al., 2017); High temperature (Burton, 1981; Hijmans, 2003; Timlin et al., 2006; Levy and Veilleux, 2007; Minhas, 2012; Bita and Gerats, 2013; Hancock et al., 2014; Rykaczewska, 2015; Tang et al., 2016; Trapero-Mozos et al., 2018); Salinity (Levy et al., 1988; Levy, 1992; Ghosh et al., 2001; Fidalgo et al., 2004; Aghaei et al., 2009; Legay et al., 2009;Queirós et al., 2009a,b, 2011; Akhtar et al., 2015; Jaarsma and de Boer, 2018)*.

The short-term shift of potato plants from ambient to elevated CO_2_ stimulate rates of net CO_2_ assimilation (Sage et al., 1989; Sicher and Bunce, 1999; Vandermeiren et al., 2002; Katny et al., 2005). However, the acclimation of photosynthetic capacity has been observed during long-term growth and development of potato plants at elevated CO_2_ concentrations as indicated by an inhibition of photosynthetic capacity that was observed under short-term CO_2_ shift (Sage et al., 1989; Ludewig et al., 1998; Sicher and Bunce, 1999; Schapendonk et al., 2000; Vandermeiren et al., 2002; Katny et al., 2005). This inhibition of photosynthetic capacity is accounted for by an accumulation of photoassimilates in the source leaves (Katny et al., 2005), which may limit P_i_ regeneration due to decreased recycling of phosphorylated intermediates (Sage et al., 1989). Additionally, the feedback inhibition of photosynthesis is associated with down-regulation of the key regulatory photosynthetic gene expression and corresponding enzyme activities, in particular, RuBisCO (Sicher and Bunce, 1999). The photosynthetic acclimation during growth at elevated CO_2_ has also been associated with stomatal closure, decrease in leaf chlorophyll content, and decreased RuBisCO activation state in the photosynthesizing source leaves (Table 1) (Sage et al., 1989; Sicher and Bunce, 1999, 2001).

Although potato plant exhibits photosynthetic acclimation to elevated CO_2_, it will increase tuber yield most from increasing CO_2_ concentrations relative to other chief food crops such as, corn, rice and wheat (Jaggard et al., 2010). So, enhancing potato yield would be crucial for food security and meet the nutrition requirements of the rising population. However, most of these high CO_2_ experiments have been conducted under ambient temperature with no water limitations. Given that the predicted rise in atmospheric CO_2_ is expected to be coupled with an increase in temperature, the yield gain achieved through high CO_2_ may be offset by yield loss due to high temperature and agricultural drought. For instance, although the projected increase in atmospheric CO_2_ is predicted to cause about 1.8% increase in global crop yields per decade over the next few decades the projected high temperature stress, water deficit and, insect and disease threats may decrease the crop yield by a 0–4% over the same period (Ziska et al., 2011; DeLucia et al., 2012; Lobell and Gourdji, 2012). Tonkaz et al. (2010) have reported a 30% decrease in winter wheat yield by a 6°C increase in growth temperature. Thus, future research on high CO_2_ experiment needs to be performed in combination with heat stress, drought and soil salinity in order to understand the effects of high CO_2_ on growth and tuber yield of potato under changing environment. In addition, it is still not understood clearly about the molecular, biochemical and physiological mechanisms of CO_2_-stimulation of yield and biomass for potato. For instance, does rising CO_2_ affect source-sink ratio? carbon and energy balance? carbon export to tubers? These are the key areas of future high CO_2_ research.

## Drought

Drought is a main abiotic stress that can strongly perturb plant performance and crop productivity mainly through inhibition of photosynthesis. Drought induced stomata closure, meant to reduce transpiration water loss and conserve plant water status, also restricts CO_2_ diffusion in the leaf making the Calvin Cycle CO_2_ substrate limited (Flexas et al., 2004; Pinheiro and Chaves, 2011; Dahal et al., 2014a). This may result in the accumulation of ATP and NADPH, since their rates of generation by photosynthetic electron transport chain do not match with their utilization by Calvin cycle. Consequently, there is an energy imbalance in the chloroplast level leading to oxidative stress and damage of cell components. Thus, in addition to stomatal limitation of photosynthesis, water deficit-sensitive biochemical machineries may also limit rates of photosynthesis during drought stress. However, the degree of contribution of stomatal limitation versus biochemical limitation to photosynthetic inhibition is still ambiguous (Flexas et al., 2004; Lawlor and Tezara, 2009; Pinheiro and Chaves, 2011; Dahal et al., 2015; Dahal and Vanlerberghe, 2017). Oliveira et al. (2012) revealed a substantial decline in wheat biomass and grain yield due to drought stress. Similarly, Hlavinka et al. (2009) reported that drought was the main cause of yield variability in eight different crops.

Because of its shallow root system, potato is considered to be the most drought-sensitive crop species. Drought stress negatively affects physiological processes involved in the tuber formation and growth (Figure 1). Potato growth and tuber yield largely depends on rainfall, consequently, even a short period of water deficit can cause a substantial loss of tuber yield and deterioration of tuber quality (Dalla Costa et al., 1997; Deblonde and Ledent, 2001). The extent of drought induced tuber yield loss, however, largely depends on the duration, severity and plant growth stage (Evers et al., 2010). Early stress is most detrimental to tuberization, bulking and tuber yield due to decreased leaf area, decreased photosynthetic rates and reduced partitioning of assimilates to tubers (Evers et al., 2010; Obidiegwu et al., 2015). If drought occurs at an early growth stage, it will substantially suppress tuber initiation, bulking and tuber yield. Drought during tuberization, causes reduction in the stolon number per stem, reflecting in lower tuber number and yield. If potato plants experience drought during tuber bulking stage, they will produce fewer and smaller sized tuber. Nevertheless, it has been suggested that stolon initiation and tuber formation are the most critical stages to drought stress. Drought-induced reduction in tuber yield is mainly associated with strong inhibition of photosynthesis due to stomatal and non-stomatal limitations (Table 1). Drought stress downregulates the expression of genes that code for chlorophyll *a*-*b* binding proteins, key regulatory photosynthetic enzymes, and sucrose biosynthetic enzymes but upregulate the expression of sucrose breaking enzymes (Hwang et al., 2011). In addition to its impact on tuber yield, drought also considerably affects numerous tuber quality parameters making them unsuitable for processing and consumption (Table 1). Drought triggers tuber defects such as, tuber cracking, hollow heart, internal brown spot, secondary growth, malformations and considerably increase a-solanine and a-chaconine glycoalkaloid content that may cause several health issues including cancer (reviewed by Wang-Pruski and Schofield, 2012). Drought is believed to induce sugar ends, a major quality disorder that causes French fries and potato chips to be dark on one end, which may lead to rejection by consumers (Thompson et al., 2008; Liu et al., 2016). Sugar end tubers are characterized by increased amount of reducing sugars, such as, glucose and fructose, at one end of the tuber. Although the actual losses accounted for by this physiological disorder is still under study, sugar ends can be costly to growers as the crop is rejected by processing industries.

## Drought Adaptation Strategies

Potato plants have evolved several strategies ranging from physiological and biochemical responses to change in gene expression and metabolic activity to combat drought stress (Table 1). These strategies enable plants either to maintain water potential by escaping the drought or develop the adaptation mechanisms to tolerate lower water potential. These strategies, however, depend largely on cultivar, growth stage and drought severity. One of the important strategies used by potato plants to survive drought stress is improvement of WUE through reduction in leaf number, leaf area and stomatal conductance as a means to minimize transpiration water loss and conserve leaf water status (Table 1) (Deblonde and Ledent, 2001; Coleman, 2008; Albiski et al., 2012). However, the associated cost will be reduced leaf surface area for photosynthesis, thus negatively impacting carbohydrate synthesis. The leaves become narrower and develop leaf hair to reduce the light absorbance and prevent photooxidative damage. Some studies have suggested an increase in root to plant biomass ratio during drought stress. Potato cultivars exhibiting higher root to shoot ratio owing to extensive and large root architecture have been found to be less susceptible to drought stress as they can increase nutrient and water uptake efficiency (Wishart et al., 2013; Villordon et al., 2014; Zarzynska et al., 2017). Zarzynska et al. (2017) tested five potato cultivars and reported a correlation between root length and area to tuber yield under drought stress. They concluded that potato cultivars with deeper root length and larger root systems exhibit increased drought tolerance. Acclimation of potato to mild drought stress can reduce yield losses under severe drought stress. Drought acclimation cycles followed by severe drought treatment reduced leaf wilting, induced thicker cuticular layer and more open stomata compared to plants without acclimation treatment (Banik et al., 2016).

Drought tolerance has also been conferred to the accumulation of compatible solutes (Rontein et al., 2002; Chen and Murata, 2008). These solutes decrease the leaf water potential without affecting turgor pressure. This will help leaf cells draw water from the soil. Accumulation of glycine-betaine has been observed in higher plants during drought, salinity and low temperature stress (Rontein et al., 2002). Using transgenic potato lines with increased betaine aldehyde dehydrogenase, an enzyme for glycine betaine biosynthesis, Zhang et al. (2011) has been able to induce drought stress tolerance in potato. In another study Vasquez-Robinet et al. (2008) revealed increased accumulation of sugar alcohol and Ambard-Bretteville et al. (2003) found elevated proline levels in potato leaves in response to drought stress. At molecular and genomic levels, drought induces expression of numerous stress-related genes that encode proteins including transcription factors and enzymes involved in drought stress tolerance (reviewed by Shinozaki and Yamaguchi-Shinozaki, 2007). The products of drought-induced genes are involved in initial stress response and in creating cellular level stress tolerance. Drought stress stimulates the synthesis of abscisic acid (ABA), a phytohormone. ABA is a well-known signaling molecules for closing stomata and inducing expression of several stress-related genes. ABA-induced expression of stress-related genes have been confirmed through exogenous ABA application. However, there are several drought-induced genes which are insensitive to exogenous ABA application suggesting that drought-related genes are governed through both ABA-dependent and ABA-independent mechanisms. Vasquez-Robinet et al. (2008) reported that drought tolerant cultivars upregulate the expression of chloroplast-localized antioxidant and molecular chaperones. The ability of potato plants to tolerate drought stress is believed to be governed by upregulation DREB1A (Dehydrin responsive element binding protein) regulons (Schafleitner et al., 2007). To support this, Watanabe et al. (2011) compared AtDREB1a transgenic potato with non-transgenic ones and observed increased drought tolerance in DREB1a transgenic lines. Transgenic potato plants overexpressing *ScCBFI* gene from *Solanum commersonii* exhibit increased drought tolerance as indicated by improved overall plant performance and extensive root development following drought stress (Pino et al., 2013).

In the past century, intensive crop breeding has been focused on selecting drought resistant cultivars by considering yield, plant phenotype, leaf morphology, osmolyte content and leaf water content as the indicators with little efforts on the rates of either photosynthesis or respiration. Although the majority of the research suggests that enhancing photosynthetic performance under drought strongly improves plant growth, its effect on tuber yield and quality is still not well understood, Thus, future research needs to be focused on understanding the photosynthetic and respiratory regulation of tuber yield and quality during drought stress. Since potato cultivation is expanding to water limited areas and the predicted climate change may further aggravate drought severity, understanding the precise mechanism of drought tolerance at the levels of molecular, biochemical and physiological is critical to improve tuber yield.

## High Temperature

As a cool weather crop potato grows well under moderate temperature in temperate regions. Temperature above the optimal is likely to inhibit plant growth and survival and hence causes reductions in tuber yield and productivity (Levy and Veilleux, 2007; Tang et al., 2018). The susceptibility of potato crops to high temperature, however, largely depends on cultivars (Tang et al., 2018), growth stage and stress duration (Ahn et al., 2004). The optimal temperature requirements for above ground plant growth and below ground tuber growth are different, the former perform well in the range of 20–25°C and the later in the range of 15–20°C (Van Dam et al., 1996). Rykaczewska (2015) tested six potato cultivars to determine whether the temperature sensitivity of potato plant is growth stage dependent and revealed that the earlier high temperature stress occurs, the more negatively it impacts the plant growth and tuber yield. Temperature above 25°C has been found to delay tuberization. It has been predicted that potato yield will decline substantially by 2055 as a consequence of global warming and drought (Holden et al., 2003). In another study Hijmans (2003) anticipates that world potato production will decline by 18–32% in the projected period of 2040–2069.

High temperature negatively impacts tuber yield and quality through inhibition of carbon synthesis and its subsequent translocation to stolon (Figure 1 and Table 1). It has been suggested that the optimum temperature for photosynthesis and biomass accumulation in potato is about 20°C and an increment of every 5°C above the optimum may decrease photosynthetic rate by 25% (Burton, 1981; Timlin et al., 2006). In another study Burton reported that temperatures higher than 30°C cause a complete inhibition of the net photosynthesis in potato (Burton, 1972). However, the response of potato plants to temperature varies across the cultivars, for instance, the commercial cultivar Russet Burbank exhibited maximum rates of photosynthesis at 24 to 30°C with photosynthetic reduction observed only at or above 35°C (Dwelle et al., 1981). Similarly, although growth at 30/20°C day/night temperature reduced tuber yield, the high temperature stimulated the rates of photosynthesis (Hancock et al., 2014). High temperature-induced inhibition of rates of photosynthesis is associated with a reduction in RuBisCO activation state, irreversible photosystem II damage and increased rates of photorespiration in many plant species including wheat, rye and canola (Long et al., 2004; Salvucci and Crafts-Brandner, 2004; Ainsworth and Rogers, 2007; Kumar et al., 2009; Dahal et al., 2012). In potato, although few studies suggest that high temperature inhibits PSII activity (Havaux, 1993), its effect on RuBisCO activity and photorespiration is still not known. Additionally, heat stress has been suggested to inhibit tuberization and tuber yield through change in assimilate partitioning and impaired sucrose translocation to tubers (Table 1). The delayed tuberization is associated with high temperature-induced inhibition of tuberization signal (Ewing, 1981), which is thought to be an ortholog of the Arabidopsis protein FLOWERING LOCUS T (FT) known as StSP6A (Navarro et al., 2011). High temperature decreases harvest index and thus, tuber weight by inhibiting the carbon export to the sink organs (tubers) from the source leaf. The assimilated carbon is accumulated to source leaves or diverted to other leaves as indicated by increased leaf dry matter (Hancock et al., 2014). It has been reported that the accumulation of carbon in the source leaves has multiple consequences including feedback limitation of photosynthesis.

High temperature negatively impacts tuber quality such as, hollow heart, tuber cracking, secondary growth, malformations. Heat stress stimulates conversion of starch to reducing sugars that triggers dark French fries (Minhas, 2012; Wang-Pruski and Schofield, 2012). High temperature induces heat necrosis in the tuber flesh deteriorating fresh market and processing quality. Moreover, high temperature triggers skin russeting (Wang-Pruski and Schofield, 2012) chain and misshapen tubers, field sprouting and decreased dry matter content in response to high soil temperature.

## High Temperature Adaptation Strategies

Plants exhibit different strategies at the physiological, morphological and molecular levels to cope with high temperature stress (see review by Bita and Gerats, 2013). One strategy that plants employ following heat stress is a considerable increase in activity of heat stress transcription factors (*HSFs*), which trigger an increase in heat shock proteins (HSPs) accumulation. *HSFs* are known to involve in governing heat stress response (HSR) and acquired thermotolerance by serving as a molecular chaperones (Pirkkala et al., 2001; Ahn et al., 2004; Koskull-Döring et al., 2007; Jingkang et al., 2008). *HSF* of higher eukaryotes is converted from an inactive monomeric to an active trimeric form following heat stress (Nover and Scharf, 1997). In higher eukaryotes, *HSF* trimerization is essential for their high-affinity binding to the heat shock element (HSE). In a genome-wide study of potato *HSFs*, Tang et al. (2016) identified a total of 27 *StHSFs* in the *Solanum tuberosum* genome and their diverse regulatory roles under adverse conditions. However, the underlying molecular mechanisms on how heat stress initiates *HSFs* trimerization for their activation and synthesize HSPs is still unknown. Trapero-Mozos et al. (2018) reported an increased expression of *HSc70* in Desiree potato cultivars at high temperature which resulted into improved tolerance to heat stress as indicated by increase in tuber yield.

The effect of high temperature associated with climate change on potato crop yield is still a major area of research. There is still little information about the growth stage most critical to temperature stress (Levy and Veilleux, 2007). The mechanism by which potatoes initially sense the changes in their surrounding temperature and consequently respond to this change at the physiological, biochemical and molecular levels is still not fully understood. Elucidation of such mechanism may offer new insights into the identification of specific characteristics that will be useful in breeding new cultivars aimed at sustaining or even enhancing potato crop productivity and quality in response to climate changes.

## Salinity

Salinity is a major threat to crop production worldwide affecting over 800 million hectares of land and representing more than 6% of the global land area (Munns and Tester, 2008). Saline soils are predominant in semi-arid and arid regions. Saline soils are usually developed through irrigation of water contaminated with salts, salt water inundation and lack of precipitation to flush out salts from the soil. The accumulation of Na^+^, Cl^-^, or SO_4_^2-^ ions in soil results in poor soil physical and chemical properties including decreased soil porosity, water permeability and soil structure. Salinity is measured in terms of electrical conductivity and osmotic potential. Climate change is predicted to raise the sea level, which may subsequently inundate agricultural soils with saline water in the coastal regions (Rahmstorf, 2007; Dasgupta et al., 2009). The expected increase in drought severity and frequency may also contribute to soil salinity through upward movement of the ground water contaminated with salts. Salinity adversely affects plant growth and development through osmotic stress in the plant. Soil containing higher dissolved solutes of salt lowers the soil water potential affecting water balance in the plant-soil continuum. In order for leaves to maintain downhill water gradient, they require to lower the water potential below the soil water potential resulting water deficit in plants. Thus, the effects of salinity is similar to those of drought stress in many aspects discussed in earlier chapter. In addition, the accumulation of ions of Na^+^, Cl^-^, or SO_4_^2-^ in cytosol causes ion toxicity and needs to be stored in the vacuole (Queirós et al., 2009a). These ions when present in higher concentrations in the cytosol, hinder enzymatic reactions, protein synthesis and plasma membrane permeability. Salt-induced injury to plant has also been associated with nutrient imbalance and oxidative stress (Queirós et al., 2009a). In sensitive species, salinity retards seedling emergence, root growth and leaf water potential leading to desiccation and early senescence. Salinity also inhibits rates of photosynthesis and alter the expression of genes associated with carbohydrate and amino acid metabolism (Legay et al., 2009). Plants have evolved several strategies to adapt to salt stress. Three main salinity tolerance strategies in plants have been suggested; (i) ion exclusion from shoot and leaves, (ii) tissue tolerance, and (iii) shoot ion-independent tolerance (Munns and Tester, 2008; Roy et al., 2014). Salinity tolerance in plants is accounted for by adjustment at the molecular, biochemical, physiological and morphological levels.

Potato is considered to be sensitive to salinity stress. Soils that are rich in salt content are detrimental to plant growth and tuber development. The magnitude of salinity effects on potato plant, however, varies with cultivars and salt levels. Salinity hastened the onset of leaf senescence, leaf yellowing and desiccation, and reduced tuber fresh and dry weight per plant (Levy et al., 1988; Levy, 1992; Akhtar et al., 2015). Jaarsma et al. (2013) also reported growth inhibition and early senescence in salt sensitive cultivars Mozart and Mona Lisa in response to salt treatment. Salinity inhibited the shoot growth and total fresh and dry biomass although the inhibition was more pronounced in salt sensitive cultivar, Concord, relative to tolerant cultivar, Kennebec (Aghaei et al., 2009). The decrease in shoot growth and biomass became severe in both cultivars with increased salt dose. Salt stress decreased leaf area, relative water content, leaf stomatal conductance and transpiration rate substantially (Fidalgo et al., 2004; Odemis and Caliskan, 2014; Akhtar et al., 2015). Salt stress also significantly inhibited rates of photosynthesis (Odemis and Caliskan, 2014; Akhtar et al., 2015). At ultrastructural level, salinity induced cell vacuolation and thylakoid swelling, and caused reduced grana stacking (Fidalgo et al., 2004; Queirós et al., 2009a). Salinity substantially increased the callus Na+ and Cl^-^ content, increased lipid peroxidation and reduced the growth rate of the callus (Queirós et al., 2007, 2011). Potato plants grown under saline conditions exhibited higher sodium content in leaves, stems, and tubers (Ghosh et al., 2001), Salinity delayed seedling emergence, and inhibited tuber growth and dry matter content, the extent of which increased with higher salt levels (Ghosh et al., 2001). Salt stress reduced the total and marketable tuber yield, which was associated with lower tuber number per plant and average tuber weight (Ghosh et al., 2001; Odemis and Caliskan, 2014). Salinity is suggested to increase the contents of water-soluble carbohydrates, starch and total non-structural carbohydrates in leaves (Ghosh et al., 2001). The decreased tuber dry matte content but increased leaf carbohydrate content may suggest that salt stress may retard carbon translocation to tuber. Salinity is also believed to increase tuber nitrogen content but decrease nitrogen reductase activity of leaf (Ghosh et al., 2001). Through cDNA microarray study, Legay et al. (2009) observed that when exposed at 150 mM NaCl potato plants down-regulate expression of genes associated with photosystem (PSII) and PSI chlorophyll synthesis and Calvin cycle enzymes.

## Salinity Adaptation Strategies

Potato plants employ several strategies to avoid salt injury. One of the key strategies used by potato plants to adapt to salt stress is to exclude Na^+^ accumulation in the cytosol of leaf cells (Queirós et al., 2009b). This can be achieved by preventing salt export from shoot to the leaves. Alternatively, the ions can be transported from cytosol to vacuole (Queirós et al., 2009b), which not only maintain the enzyme activity in the cytosol but also adjust cell water potential. Jaarsma and de Boer (2018) studied the gene expression, corresponding protein levels and activity associated with vacuolar proton pumps and the Na^+^/H^+^ antiporters in salt tolerant (cv. Desiree and salt sensitive (cv. Mozart) potato cultivars subject to 60 mM NaCl. Their result revealed that potato plant achieve salt tolerance by accumulating ions in the vacuole through vacuolar proton pump-driven Na^+^ transport across the tonoplast in exchange for H^+^. The activity of V-H^+^-ATPase and the V-H^+^-PPase, and the corresponding protein levels were inhibited in both cultivars following salt treatment (Jaarsma and de Boer, 2018). However, upon salt treatment, the V-H^+^-PPase activity and protein amount were higher in resistant versus sensitive cultivars, which is likely to confer salt tolerance in Desiree. Furthermore, these cultivars responded differentially to H^+^ pump activity such that the decline in H^+^ pump activity was less pronounced in the salt tolerant Desiree relative to sensitive Mozart. This was reflected to higher Na^+^/H^+^ exchange activity and increased Na^+^ affinity in Desiree than in Mozart. The authors suggest that the higher capacity for Na^+^/H^+^ exchange and affinity for Na^+^ uptake confers salt tolerance in Desiree. These results were consistent with previous study, which revealed that salt tolerance in potato is associated with Na^+^ sequestration into vacuole through increase Na^+^/H^+^ antiport activity and pH gradient across the tonoplast (Queirós et al., 2009b). Jaarsma et al. (2013) grew six potato cultivars varying in salt tolerance under hydroponic system with different NaCl levels. Their study suggest that the Na^+^ accumulation in shoot is positively correlated with salt tolerance with tolerant cultivars capable of inhibiting Na^+^ export from shoot to leaves.

Similar to drought adaptation strategy potato plants adapt to salinity stress through osmotic adjustment by accumulating compatible solutes in the cytosol (Fidalgo et al., 2004). Salinity decreased leaf water potential leading to reduced cell turgidity, growth retardation and tuber yield loss (Levy et al., 1988). Accumulation of compatible solutes is believed to maintain cell turgor pressure without affecting cytosolic enzymatic reactions. This notion is supported by the fact that the proline, a well-known compatible solute, content of Desiree leaves increased by 3.5-fold and 11-fold at 100 and 200 mM NaCl, respectively, compared to untreated controls (Fidalgo et al., 2004). Similar result was obtained following short-term exposure or long-term growth under saline conditions (Queirós et al., 2011). However, recently Odemis and Caliskan (2014) reported that the foliar application of proline was not effective to minimize the effects of salinity on several physiological characteristics and yield parameters. Thus, effects of proline on salt stress tolerance needs further study. Potato plants under salinity stress adapt to osmotic stress by reducing leaf area and advancing premature leaf senescence to preventing transpirational water loss. Potato plants also avoid salt injury by shortening growth cycle such that the early-maturing exhibits lower tuber yield loss compared to late-maturing cultivars (Levy et al., 1988). Legay et al. (2009) reported that potato plants adapt to osmotic stress through induction of transcription factors mediated by ABA-dependent or ABA-independent pathways. Salt stress induced the synthesis of ABA in both leaf and xylem sap (Akhtar et al., 2015). ABA is a well-known signaling molecules for closing stomata and inducing expression of several genes involved in stress tolerance including salinity.

Another strategy used by potato plant to adapt salt stress is to activate reactive oxygen species (ROS) scavenging pathways. Salt stress generates ROS in the form of singlet oxygen, superoxide, peroxides, hydroxyl radical, which are detrimental to normal cell function as they cause oxidative damage to protein, nucleic acids, lipids among others. Scavenging of ROS is, therefore, critical for plants to survive salt stress. To understand the importance of ROS scavenging in potato, Aghaei et al. (2009) analyzed antioxidant enzyme activities and ion content in tolerant (cv. Kennebec) and sensitive (cv. Concord) cultivars subject to various salinity treatments. They observed a decrease in activity of antioxidant enzymes; ascorbate peroxidise, catalase and glutathione reductase in the shoot of salt sensitive cultivar, Concord, in response to NaCl treatment. In contrast, the activity of all these enzymes were upregulated in salt-tolerant cultivar, Kennebec, following NaCl treatment. This was reflected to higher plant biomass in Kennebec as compared to Concord under salinity stress, suggesting that increased antioxidant enzyme activity makes Kennebec tolerant to salt stress. In a similar experiment, Fidalgo et al. (2004) reported a significant increase in superoxide dismutase activity but decrease in catalase activity with no change in ascorbate peroxidase activity in Desiree at 100 and 200 mM NaCl treatment relative to control. Queirós et al. (2011) also observed a decrease in catalase activity and no differences in ascorbate peroxidise activity in salt-treated versus control potato plants. Although the total superoxide dismutase, ascorbate peroxidase, and glutathione reductase activities decreased following salt treatment, the amount of ascorbate and reduced glutathione increased in salt treated versus control plants (Queirós et al., 2011).

## Perspectives for Yield Improvement and Stress Tolerance

Potato tuber mainly consists of carbohydrates assimilated through photosynthesis. Photosynthetic energy conversion efficiency of potato is only 0.0411 (Slattery and Ort, 2015), which is less than 0.046 average value for C_3_ species (Figure 2). Therefore, there is a great potential to enhance potato tuber yield through improved photosynthetic efficiency. Future research needs to identify and characterize potato cultivars exhibiting a higher rates of photosynthesis, which can be used effectively in breeding new cultivars with a higher yield potential. Moreover, the biotechnological approaches that could improve RuBisCO activity, lower photorespiratory loss of carbon and non-photochemical quenching of absorbed light energy may be remarkable to enhance photosynthetic yield potential (Zhu et al., 2008, 2010).

The predicted rise in atmospheric CO_2_ level is expected to increase crop yield and biomass through stimulation of photosynthesis and suppression of photorespiration albeit the enhancement is crop species dependent (Long et al., 2006; Dahal et al., 2012). However, an important concern with increase in atmospheric CO_2_ is concomitant increase in heat stress and associated water deficit and biotic stress. Thus, although the projected rise in the atmospheric CO_2_ is expected to increase yield potential in certain crop species, the yield deterioration due to climate change associated heat stress, drought stress and biotic stress can outpace the benefit achieved by any increase in atmospheric CO_2_. It is one of the challenges for plant breeders and agriculturists to maintain yield stability through improved tolerance to climate change associated stresses such as drought, low and high temperature in future (Powell et al., 2012). Potato crop, like most other crop species, is highly prone to abiotic stress particularly, high temperature, drought and soil salinity. In order to maintain a sustainable potato production under climate change, we must identify best cultivation practices and develop heat, drought, insect and pathogen tolerant cultivars that can best adapt to the changing environment. It is one of the challenges for crop scientists to pinpoint means of improving crop yield and quality at high CO_2_, high temperature and drought stress as well as biotic stress. One important approach is to understand stress-related molecular, biochemical and physiological markers that can be used to develop screening procedures for selection of crop cultivars that can better adapt to sub-optimal growth conditions. The mechanism by which potato plants initially sense the changes in their surrounding CO_2_, temperature, water status, soil salinity and biotic stress and consequently respond to these changes at the molecular, biochemical and physiological levels is still under infancy. Future research needs be concentrated on the identification of signaling molecules, target genes and their networks that govern plant phenotypic and metabolic plasticity in response to elevated CO_2,_high temperature and water deficit. Elucidation of such mechanism may provide new insights into the identification of specific characteristics that may be useful in breeding programs aimed at (1) developing new potato cultivars with enhanced yield potential and (ii) developing potato germplasm with adaptation to climate change. This will help in sustaining or even enhancing potato productivity under predicted climate change.

## Conclusion

Enhancing potato productivity is important to meet the global food demand of an increasing population. However, potato plant growth and tuber yield is constrained by high temperature, water limitation, soli salinity, and insect and pathogen threats. Climate change will likely further aggravate tuber yield losses by intensifying potato plant’s exposure to these stress conditions. Hence, there is an urgent need to adapt to the new cropping challenges by developing heat, drought, insect and pathogen-tolerant crop cultivars that are appropriately engineered for the changing environment. Improving potato plant adaptability to environmental stresses under increasing CO_2_ is one of the most important and challenging targets. This challenge can be approached through the identification of stress-related traits at the physiological, biochemical and molecular levels and their deployment in new cultivars. In addition, the development of new knowledge and techniques on “omics-driven” high-throughput approaches is crucial to advance screening procedures for breeding potato cultivars aimed at improving plant adaptability and tuber productivity in response to climate change.

## Author Contributions

KD wrote the article. X-QL, HT, AC, and BB commented on the writing and participated in revision.

## Conflict ofInterest Statement

The authors declare that the research was conducted in the absence of any commercial or financial relationships that could be construed as a potential conflict of interest.
